# Acute obstructive pancreatitis after pancreas-sparing total duodenectomy in a patient with pancreas divisum: a case report

**DOI:** 10.1186/s40792-016-0255-1

**Published:** 2016-11-05

**Authors:** Shimpei Otsuka, Teiichi Sugiura, Katsuhiko Uesaka

**Affiliations:** Division of Hepato-Biliary-Pancreatic Surgery, Shizuoka Cancer Center, 1007 Shimo-Nagakubo, Sunto-Nagaizumi, Shizuoka 411-8777 Japan

**Keywords:** Pancreas-sparing total duodenectomy, Pancreas divisum, Obstructive pancreatitis

## Abstract

**Background:**

Pancreas-sparing total duodenectomy (PSTD) is an ideal recommended procedure for patients with multiple duodenal adenomas or early duodenal cancer. We herein report a rare but serious complication of PSTD.

**Case presentation:**

A 20-year-old woman with duodenal adenocarcinoma underwent PSTD. On postoperative day one, she complained of severe abdominal pains. Her serum amylase and serum pancreatic amylase levels were extremely elevated (Amy, 1296 IU/L; P-Amy, 1273 IU/L). With contrast enhanced CT, acute obstructive pancreatitis with pancreas divisum due to the ligation of the dorsal pancreatic duct was highly suspected. An emergency operation was performed to relieve the pancreatic duct obstruction, and an additional anastomosis between the dorsal pancreatic duct and jejunum was performed. The patient’s postoperative course was mostly uneventful, and her discomfort improved immediately.

**Conclusion:**

When we perform pancreas-sparing total duodenectomy, some form of pancreatography is necessary to exclude pancreas divisum.

## Background

Pancreas-sparing total duodenectomy (PSTD) is an ideal recommended procedure for patients with multiple duodenal adenomas or early duodenal cancer [1, 2]. It may have some advantages for pancreatoduodenectomy due to the preservation of pancreatic function. But a specific complication of this procedure is not well known. We herein report a rare but serious complication of PSTD.

## Case presentation

**Fig. 1:**
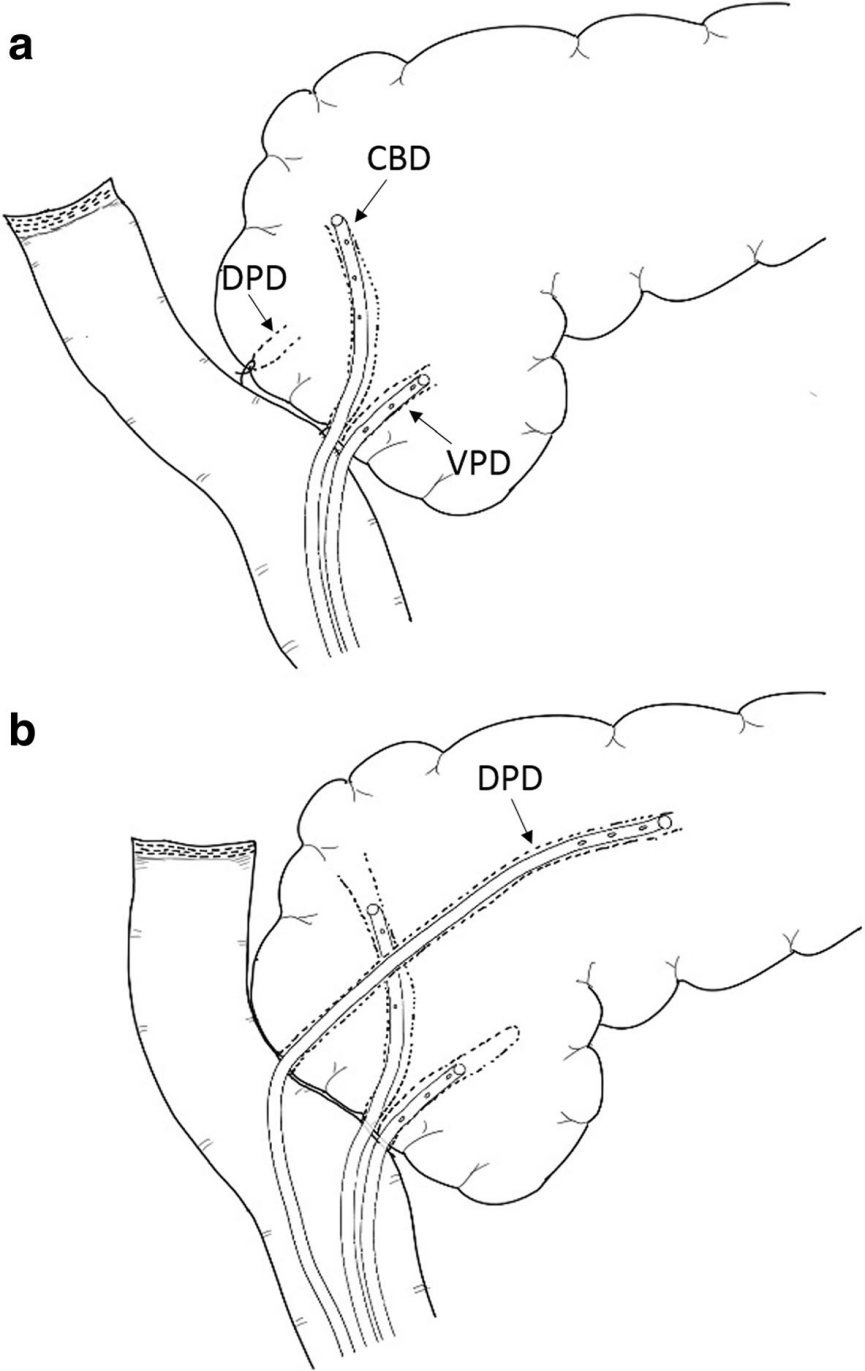
The operating protocol. **a** For the first operation, anastomosis between the bilio-pancreatic common duct and jejunum with two external stents was performed. The dorsal pancreatic duct was ligated. **b** For the second operation, additional anastomosis between the dorsal pancreatic duct and jejunum was performed. *CBD* common bile duct, *VPD* ventral pancreatic duct, *DPD* dorsal pancreatic duct

**Fig. 2:**
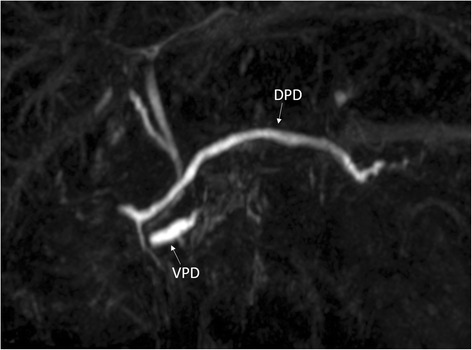
Magnetic resonance cholangiopancreatography demonstrated no communication between the dorsal pancreatic duct and the ventral pancreatic duct: pancreas divisum

A 20-year-old woman had been treated at our hospital for 3 years due to familial adenomatous polyposis with Spigelman classification of stage IV. Periodic upper gastrointestinal endoscopy revealed multiple duodenal adenomas around the duodenal papilla. Endoscopic biopsy showed that two of the lesions were adenocarcinomas. The tumors were diagnosed as in situ cancers with endoscopic examination including endoscopic ultrasonography. To remove all duodenal lesions but not lymph node, she underwent laparoscopic-assisted PSTD. The stomach was divided at 2 cm from the pylorus. The total duodenum was fully dissected from the head of the pancreas. The dorsal pancreatic duct seemed to be normal size and it was ligated and divided. Plasty of the ventral pancreatic duct and bile duct was performed to the common trunk. Anastomosis between bilio-pancreatic common duct and jejunum with two external stents, and gastrojejunostomy (Roux-en-Y) was performed by small (7 cm) laparotomy (Fig. 1a). On postoperative day (POD) 1, she complained of severe abdominal pains. Her serum amylase (Amy) and serum pancreatic amylase (p-Amy) levels were extremely elevated (Amy, 1296 IU/L; P-Amy, 1273 IU/L), respectively. Contrast enhanced computed tomography on POD 2 revealed peripancreatic inflammation and a dilated pancreatic duct. There was no communication between the dorsal duct and the ventral duct (dominant dorsal duct sign). Considering these findings, acute obstructive pancreatitis with pancreatic divisum due to the ligation of the dorsal pancreatic duct was highly suspected. An emergency operation was performed to relieve the pancreatic duct obstruction. The ligation of the dorsal pancreatic duct was detected and released. Additional anastomosis between the dorsal pancreatic duct and jejunum (duct-to-mucosa anastomosis using incomplete external drainage of pancreatic duct) was performed (Fig. 1b). The patient’s postoperative course was mostly uneventful (no pancreatic fistula), and her discomfort improved immediately. She was discharged 28 days after the first operation. Magnetic resonance cholangiopancreatography (MRCP) 6 months after operation clearly demonstrated pancreas divisum (Fig. 2).

### Discussion

Pancreas divisum is the most common congenital variant of pancreatic duct development and occurs in 0.5–17.6 % of individuals [3]. In such patients, the dorsal pancreatic duct secretes most of the pancreatic juice and its occlusion leads to obstructive pancreatitis.

Pancreatoduodenectomy (PD) is a common procedure for duodenal lesions, but its short- and long-term morbidity cannot be ignored. In contrast to PD, PSTD may have an advantage with regard to the postoperative pancreatic endocrine and exocrine function. Previous report showed no necessity of postoperative oral pancreatic enzyme supplementation in PSTD cases (0 %) compared to PD cases (75 %) with matched-pairs analysis [4]. However, when PSTD is planned, attention should be paid to the presence of pancreas divisum. If the divisum is recognized, double duct-to-mucosa anastomosis, invagination anastomosis, or conventional pancreatoduodenectomy is needed [2, 5].

In our institution, solitary mucosal (in situ) cancer of duodenum was indication of endoscopic submucosal dissection (ESD) because lymph node dissection would not be necessary. Multiple mucosal cancers which are difficult to treat by ESD are considered as indication of PSTD. On the other hand, advanced duodenal cancer which invades more than submucosal layer is considered as indication of pancreatoduodenectomy because systemic lymph node dissection is necessary.

Endoscopic retrograde pancreatography (ERP) displays high accuracy in the diagnosis of pancreas divisum [6]; however, the procedure is considered to be too invasive for preoperative screening. Multi-detector row computed tomography (MDCT) is a non-invasive procedure that has a high diagnostic ability when the pancreatic duct is visualized (sensitivity 90 %, specificity 97 %) [7]. MRCP is also a non-invasive procedure that can be used to diagnose the anatomy of pancreatic duct. In particular, secretin-stimulated MRCP displays high ability in the diagnosis of pancreas divisum (sensitivity 73.3 %, specificity 96.8 %) [8]. These two non-invasive procedures (MDCT and MRCP) should be performed preoperatively in PSTD cases. When the divisum cannot be excluded preoperatively, ERP [6] or intraoperative direct pancreatography [2] should be performed.

In the present case, a sufficient preoperative assessment of the pancreatic duct could not be performed. Although preoperative MDCT showed slight dilation of dorsal pancreatic duct, we did not notice such finding preoperatively due to the lack of knowledge. Therefore, we did not carry out MRCP because we are never afraid of such a serious complication relating PSTD in patient with pancreas divisum. Considering our bitter experience, it is of importance to have a thorough knowledge about the benefit and risk of newly performed procedure.

## Conclusions

When we perform PSTD, some form of pancreatography is necessary to exclude pancreas divisum.

## Abbreviations

MDCT: Multi-detector row computed tomography
MRCP: Magnetic resonance cholangiopancreatography
PD: Pancreatoduodenectomy
PTSD: Pancreas-sparing total duodenectomy

## References

[1] Chung RS, Church JM, vanStolk R (1995). Pancreas-sparing duodenectomy: indications, surgical technique, and results. Surgery.

[2] Stauffer JA, Adkisson CD, Riegert-Johnson DL, Goldberg RF, Bowers SP, Asbun HJ (2012). Pancreas-sparing total duodenectomy for ampullary duodenal neoplasms. World J Surg..

[3] Liao Z, Gao R, Wang W, Ye Z, Lai XW, Wang XT (2009). A systematic review on endoscopic detection rate, endotherapy, and surgery for pancreas divisum. Endoscopy..

[4] Muller MW, Dahmen R, Koninger J, Michalski CW, Hinz U, Hartel M (2008). Is there an advantage in performing a pancreas-preserving total duodenectomy in duodenal adenomatosis?. Am J Surg..

[5] Sarmiento JM, Thompson GB, Nagorney DM, Donohue JH, Farnell MB (2002). Pancreas-sparing duodenectomy for duodenal polyposis. Arch Surg..

[6] Lehman GA, Sherman S (1998). Diagnosis and therapy of pancreas divisum. Gastrointest Endosc Clin N Am..

[7] Soto J, Lucey B, Stuhlfaut J (2005). Pancreas divisum: depiction with multi-detector row CT1. Radiology..

[8] Mosler P, Akisik F, Sandrasegaran K, Fogel E, Watkins J, Alazmi W (2012). Accuracy of magnetic resonance cholangiopancreatography in the diagnosis of pancreas divisum. Dig Dis Sci..

